# The *ATP6V1B2* DDOD/DOORS-Associated p.Arg506* Variant Causes Hyperactivity and Seizures in Mice

**DOI:** 10.3390/genes14081538

**Published:** 2023-07-27

**Authors:** Justine Rousseau, Samuel Boris Tene Tadoum, Marisol Lavertu Jolin, Thi Tuyet Mai Nguyen, Norbert Fonya Ajeawung, Ann M. Flenniken, Lauryl M. J. Nutter, Igor Vukobradovic, Elsa Rossignol, Philippe M. Campeau

**Affiliations:** 1 CHU Sainte-Justine Research Centre, University of Montreal, Montreal, QC H3T 1C5, Canadanorbert_fon@yahoo.com (N.F.A.); elsa.rossignol@umontreal.ca (E.R.); 2 Lunenfeld-Tanenbaum Research Institute, The Centre for Phenogenomics, Toronto, ON M5T 3H7, Canada; 3 The Hospital for Sick Children, The Centre for Phenogenomics, Toronto, ON M5T 3H7, Canada

**Keywords:** *Atp6v1b2*, R506*, mice, locomotor hyperactivity, diminished anxiety-related behavior, epilepsy

## Abstract

The vacuolar H^+^-ATPase is a multisubunit enzyme which plays an essential role in the acidification and functions of lysosomes, endosomes, and synaptic vesicles. Many genes encoding subunits of V-ATPases, namely *ATP6V0C, ATP6V1A, ATP6V0A1,* and *ATP6V1B2*, have been associated with neurodevelopmental disorders and epilepsy. The autosomal dominant *ATP6V1B2* p.Arg506* variant can cause both congenital deafness with onychodystrophy, autosomal dominant (DDOD) and deafness, onychodystrophy, osteodystrophy, mental retardation, and seizures syndromes (DOORS). Some but not all individuals with this truncating variant have intellectual disability and/or epilepsy, suggesting incomplete penetrance and/or variable expressivity. To further explore the impact of the p.Arg506* variant in neurodevelopment and epilepsy, we generated *Atp6v1b2*^emR506*^ mutant mice and performed standardized phenotyping using the International Mouse Phenotyping Consortium (IMPC) pipeline. In addition, we assessed the EEG profile and seizure susceptibility of *Atp6v1b2*^emR506*^ mice. Behavioral tests revealed that the mice present locomotor hyperactivity and show less anxiety-associated behaviors. Moreover, EEG analyses indicate that *Atp6v1b2*^emR506*^ mutant mice have interictal epileptic activity and that both heterozygous (like patients) and homozygous mice have reduced seizure thresholds to pentylenetetrazol. Our results confirm that variants in *ATP6V1B2* can cause seizures and that the *Atp6v1b2*^emR506*^ heterozygous mouse model is a valuable tool to further explore the pathophysiology and potential treatments for vacuolar ATPases-associated epilepsy and disorders.

## 1. Introduction

Epilepsy is characterized by excessive or hypersynchronous neuronal activity across brain networks, resulting in recurrent and unprovoked seizures [1,2,3,4]. More than 100 genes have been associated with epilepsy [5,6,7,8]. The most frequently associated genes, and the first group to be recognized as genetic cause of epilepsy, encode ion channels (with variants causing channelopathies) [5,9]. More recently, other genes regulating synaptic transmission or, for example, interneuron function, have emerged as causal genes [10]. These include vacuolar ATPase subunits involved in neurotransmitter recycling or vesicular release (*ATP6V1B2*, *ATP6V1A*, *ATP6V0C*, *ATP6V0A1*).

Dominant variants in *ATP6V1B2* [MIM 606939], encoding a vacuolar ATPase, have been associated with three different syndromes, namely Zimmermann–Laband syndrome type 2 (ZLS2 [MIM: 616455]) [11], congenital deafness with onychodystrophy syndrome (DDOD [MIM: 124480]) [12,13,14,15] and deafness with onychodystrophy, osteodystrophy, intellectual disability (ID) with or without seizures syndrome (DOORS [MIM: 220500]) [16]. The *ATP6V1B2* nonsense variant p.R506*, generating a −6 amino acid truncated protein, causes DDOD syndrome usually without seizures [12,13,14,15,17] and DOORS usually with seizures [14,16]. While one of the *ATP6V1B2* missense variants, p.R485P, causes ZLS2 without epilepsy [11], individuals with missense variants p.E374Q/G or p.L398P develop epilepsy without other classic clinical features observed in ZLS2 or DDOD syndrome [17,18]. Finally, another nonsense variant predicted to escape nonsense-mediated decay (p.K489*) causes ZLS2 with epilepsy [19]. At present, there is no clear correlation between the position of variants and the resultant syndrome, nor to the risk of epilepsy. However, variants resulting in loss of ATP6V1B2 C-terminal amino acids seem to be more deleterious and more frequently associated with seizures than missense variants.

*ATP6V1B2* encodes the V1B2 subunits of the vacuolar H^+^-ATPase (V-ATPase), which plays an essential role in organelle acidification and vesicular transport [20,21]. The V-ATPase is made of a cytoplasmic 8 subunit V1 domain that catalyzes the hydrolysis of ATP, and a transmembrane 6 subunit V0 domain that allows proton translocation. In *Drosophila*, *Atp6v1b2* knockdown results in reduced lysosomal acidification and acidification-independent autophagosome formation and fusion [22], which both lead to harmful accumulation of cellular waste. In mice, the complete loss of ATP6V1B2 results in embryonic lethality prior to organogenesis (https://www.mousephenotype.org/data/genes/MGI:109618, accessed on 25 July 2023). Mice with conditional loss of ATP6V1B2 in the cochlea [12] or insertion of the human p.R506* variant [23] are viable. As expected, both mouse models develop hearing loss, although this occurs after the neonatal period. The acquired hearing loss was associated with abnormal lysosomal degradation and the accumulation of large autophagosomes in type II spiral ganglion (SNG) neurons causing apoptosis and subsequent loss of outer hair cell (OHC) connections [23]. Decreased lysosomal acidification was also observed in vitro in mammalian cells overexpressing ATP6V1B2 R506* compared to the one overexpressing the WT protein [12]. Altogether, these results indicates that the p.R506* variant causes a loss of function either as a result of a dominant negative effect or haploinsufficiency.

To characterize the function of the *ATP6V1B2* p.R506* variant in brain circuits, we generated *Atp6v1b2*^emR506*^ mice by CRISPR/Cas9-mediated gene editing. Since this mutation is associated with a range of neurological deficits in humans, with intellectual deficiency and, in some cases, epilepsy, we conducted neurophenotyping using the International Mouse Phenotyping Consortium (IMPC) pipeline [24] and performed baseline video-EEG monitoring. We further assessed the susceptibility of these mice to drug-induced seizures upon exposure to pentylenetetrazol (PTZ).

## 2. Materials and Methods

### 2.1. Generation of Atp6v1b2^emR506*^ Mice

The human variant NM_001693.3: c.1516C>T, p.R506* was introduced into the mouse genome using the CRISPR/Cas9 method. The mice were generated by the McGill Integrated Core for Animal Modeling (MICAM; McGill University, Montreal, Quebec, Canada). Briefly, the sgRNA (Synthego), Cas9 protein (IDT, catalog 1081058), and ssODN (ultramer, IDT) were microinjected into the pronucleus of C57BL/6N mouse zygotes with respective concentrations of 50:50:30 ng/μL. Embryos were subsequently implanted in CD-1 pseudopregnant surrogate mothers according to standard procedures approved by the McGill University Animal Care Committee (UACC). Oligonucleotides used were mAtp6v1b2-gRNA: 5′-CTCGAGGGTAAAATTCGCTC-3′ and ssODN: 5’-ATGAGACTTTGGACATTGGCTGGCAGTTGCTTCGAATCTTCCCCAAAGAAATGCTGAAGAGA.

ATCCCTCAGAGCACCCTGAGCGAATTTTATCCATAAGATTCCGCAAAACACTAGTAGCTGCTGCTGCTTGTGTGGCGTGACCCTCTTGTGAA-3’. After weaning, the mice were transferred to the Recherche du Centre Hospitalier Universitaire (CR-CHU) of Sainte-Justine Hospital. Mice were backcrossed for at least 3 generations to C57BL/6NJ (Jackson). The presence of the variant was confirmed by Sanger sequencing. Mouse husbandry and experiments at CR-CHU were conducted according to the approved animal user 732-NAGANO protocol no. 2021-3228 by the Coordonnatrice du Comité Institutionnel des Bonnes Pratiques Animales en Recherche (CIBPAR). This committee is following the guidelines of the Canadian Council on Animal Care (CCAC). All procedures involving animals at The Centre for Phenogenomics (TCP) were performed in compliance with the Animals for Research Act of Ontario and the Guidelines of the CCAC. The Animal Care Committee at TCP reviewed and approved all procedures conducted on animals at TCP which were performed under protocols 0275 and 0279.

### 2.2. Mice Phenotyping

Mice were sent to The Centre for Phenogenomics (TCP) for characterization in the International Mouse Phenotyping Consortium (IMPC) pipeline [24]. On arrival at TCP, sperm from heterozygous males was cryopreserved using previously published protocols [25,26]. Mice were subsequently rederived by IVF and embryo transfer using frozen-thawed *Atp6v1b2*^emR506*^ sperm and oocytes from C57BL/6NCrl females. Heterozygous mice were intercrossed to generate cohorts for phenotyping. Mice were genotyped using allelic discrimination on a Viia7 in multiplex with two primers flanking the variant site (snpF: CCCAAAGAAATGCTGAAGAGA and snpR2: GAAACAGAACCAGTACTTCACAAG) and two locked nucleic acid probes, one for the wild-type (WT) sequence (CCCT + C + G + AGA + CTC) and one for the mutant sequence (TA + TCCA + T + A + AGATT + C + CG) (IDT, Coralville, IA, USA). DNA was prepared from tissue biopsies using KAPA express extract KK7100 (Millipore Sigma, Burlington, MA, USA), diluted 1:2 with nuclease-free water and 2 µL diluted DNA used in a 10-µL PCR with 0.625 µM each primer and 0.312 µM each probe using the recommended protocol for PerfeCTa qPCR ToughMix Quanta BioSciences 95114-012 (QuantaBio, Beverly, MA, USA) or TaqPath ProAmp Master Mix A30871 (Thermo Fisher Scientific, Waltham, MA, USA).

Mice were housed at a density of 2 to 5 mice per cage under specific-pathogen-free conditions in individually ventilated cages Tecniplast Sealsafe Plus (Tecniplast, Buguggiate, VA, Italy), overall dimensions [L × W × H]: 199 × 391 × 160 mm). Envigo Tekland 2918S diet (Envigo, Indianapolis, IN, USA), quarter-inch corn cob bedding, and environmental enrichment of shredded paper (EnviroPak, Lab Supply, Durham, NC, USA) were included in the cage with food and water provided ad libitum. Room temperature was 21 to 22 °C and regulated at 30–55% humidity. Detailed phenotyping protocols can be found at International Mouse Phenotyping Resource of Standardised Screens (IMPReSS) (https://www.mousephenotype.org/impress/index, accessed on 25 July 2023). When the number of homozygous (HOM) mice was insufficient to meet minimum housing density or when WT littermates were required for phenotyping, heterozygous (HET) or WT littermates were co-housed with HOM mice. The control group used for analyses included WT littermates as well as WT C57BL/6NCrl mice phenotyped through the IMPC phenotyping pipeline within a fixed time window (~10 months) corresponding to the testing of the HOM mice. A total of 22 mice for each sex were used as controls (littermates and unrelated wildtype mice). For HOM mutant mice, 10 males and 7 females were phenotyped. The following tests were performed: weekly body weight measurements—Weeks 4–16, Open Field—Week 9, SHIRPA—Week 9, Grip Strength—Week 9, Light-Dark—Week 10, Pre-Pulse Inhibition (PPI)—Week 10, Fear Conditioning—Week 11, ECG—Week 12, IPGTT—Week 13, Auditory Brainstem Response (ABR)—Week 14, X-ray—Week 14, Body Composition—Week 14, Slit Lamp—Week 15, Ophthalmoscope—Week 15, Hematology—Week 16 and Blood Biochemistry—Week 16 (Supplementary Figure S1).

### 2.3. Video-EEG Recordings

Intracranial video EEG recordings were conducted in P90-P110 animals. We implanted unipolar stainless-steel electrodes (0.005-inch diameter E363/3/SPC, Plastic One) adapted to a Delrin 7 channels pedestal (MS7P, Plastic One) under isoflurane anesthesia (2% in O_2_, 0.8 L/min), while holding core body temperature at 36.5 °C. Trepanation holes were drilled over the somatosensory cortex (S1) and hippocampal CA1 areas and the electrodes were inserted in the target areas (S1: AP/ML/DV: −1.0/±1.5/−1 mm; CA1: −1.5/±1.5/2.0 mm to Bregma). Reference and ground electrodes were inserted separately over the cerebellum. The head mount was fixed to the skull using dental cement (Motloid) and a frontal screw. The animals were allowed to recover progressively in a temperature-controlled recovery cage and were returned to their home cage environment once fully awake. We provided buprenorphine (0.10 mg/kg IP every 12 h for 48 h) for post-op analgesia and the mice were monitored daily following the surgeries to ensure their well-being and recovery. Forty-eight hours after surgery, the mice were transferred to individual housing/recording home cages made of transparent Plexiglas, with ad libitum access to water and food. The head mounts were connected to a pre-amplifier and to the recording unit using flexible wires allowing for free movement of the animals in their cages. Video-EEGs were recorded continuously (24 h/24 h) for 72 h at 2000 Hz acquisition speed, filtered at HP 0.1 Hz and LP 300 Hz and digitized using the Cervello NeuroMed acquisition system (Blackrock Microsystems). All recordings were conducted in a Faraday cage to minimize noise. Traces were visualized and annotated manually for movement artefacts. Epileptic activity was defined as spontaneous continuous spike or spike-wave discharges (>200 mV) lasting more than 4 s, with simultaneous behavioral changes (arrest of activity, tonic posture, tonic-clonic or myoclonic movements) on video recordings. Seizures were quantified in terms of frequency, duration, and seizure severity, according to the modified Racine scale, as previously described [27]. Interictal epileptic activity was defined as epileptic discharges lasting less than 4 s.

### 2.4. Seizure Threshold to Pentylenetetrazol (PTZ)

After 72 h of baseline recordings, the mice were exposed to a continuous intravenous perfusion of pentylenetetrazol (PTZ) (0.01 mg/mL in saline, administered via the caudal vein) at 0.1 mL/min until the first myoclonic twitch was observed together with seizure activity on EEG. The PTZ infusion was then stopped, and the tubing was disconnected from the caudal vein needle. Blood backflow in the needle confirmed adequate placement of the needle. Monitoring with continuous video-EEG was pursued for 3 h post-infusion. We quantified the time latency to the first seizure and the minimal dose of PTZ required to induce seizures (mg/kg), reflecting the pharmacological seizure threshold. Mean values were compared between mice genotypes using a One-way ANOVA (GraphPad). The mice were sacrificed at the end of the recordings, using Pentobarbital injections (50 mg/kg, i.p.), and were perfused with an intracardiac infusion of ice cold 4% PFA in PBS. The brains were micro-dissected, extracted, and post-fixed for 1 h in 4% PFA in PBS at 4 °C, cryopreserved in 30% sucrose at 4 °C overnight and embedded in O.C.T. Compound 4585 (Thermo Fisher Scientific, Waltham, MA, USA) for conservation at −20 °C. The brains were subsequently sectioned in 18 μm cryosections, processed for DAPI staining as previously described. Images were acquired with a Leica SP8 confocal microscope and analyzed to confirm accurate electrode placement.

### 2.5. Statistical Analysis

All statistical analyses were performed using GraphPad. Two-way ANOVA with repeated measures was used for the body weight analysis. For all the other analyses, a One-way ANOVA test was conducted first. When no significant difference was observed between male and female, data were pooled together, and either a Student *t*-test, Welch’s test, or a Mann–Whitney test was performed. Statistical significance is indicated as follow: ns = non-significant, * = *p* value ≤ 0.05, ** = *p* value ≤ 0.01, *** = *p* value ≤ 0.001 and **** = *p* value ≤ 0.0001.

## 3. Results

### 3.1. Atp6v1b2^emR506*^ Mice Present Locomotor Hyperactivity

**Figure 1:**
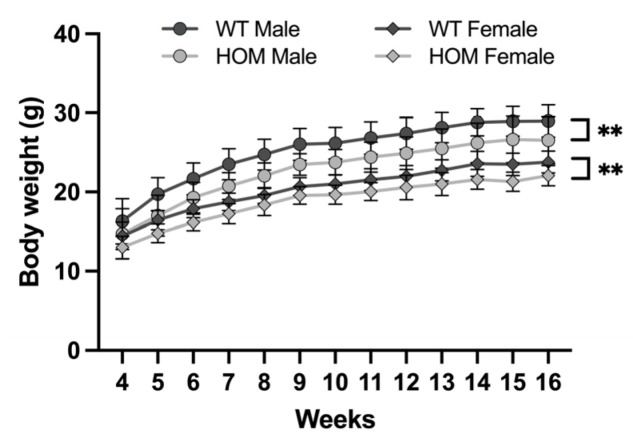
*Atp6v1b2*^emR506*^ HOM mice have a decreased body weight. Body weight measurements from week four to sixteen. Results are expressed as the mean ± SD. *n* = 22 for WT male and female, *n* = 10 and *n* = 7 for HOM male and female respectively. Two-way ANOVA with RM was applied. Genotype main effect for male: F(1, 30) = 11.20, *p* = 0.0022 and female: F(1, 27) = 9.750, *p* = 0.0042. ** *p* value ≤ 0.01.

*Atp6v1b2*^emR506*^ HOM mice are viable and were born at the expected Mendelian ratio. The mice were enrolled in The Centre for Phenogenomics (TCP) early adult phenotyping pipeline in accordance with the International Mouse Phenotyping Consortium (IMPC). Apart from the body weight, where both male and female homozygous mutants showed significantly lower body weight compared to WT (Figure 1), no other morphological abnormalities were observed.

**Figure 2:**
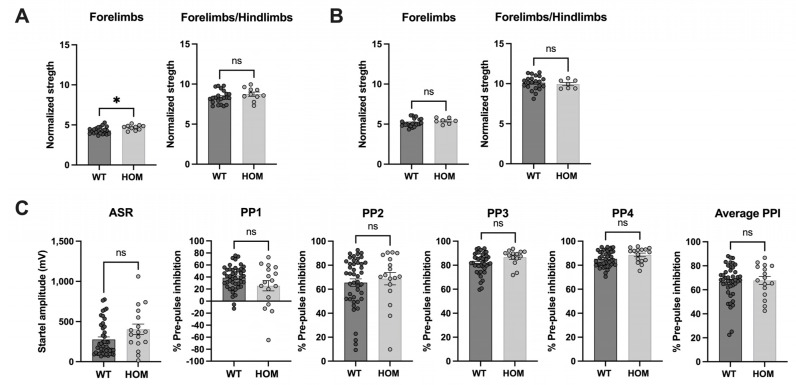
*Atp6v1b2*^emR506*^ HOM mice show normal neuromuscular and sensorimotor gating. Forelimbs and Forelimbs/Hindlimbs strength of males (**A**) and females (**B**) normalized to body weight during the strength test. Student’s unpaired t test was applied. (**C**) Percentage of Pre-Pulse inhibition following pre-pulse at 70 dB (PP1) to 85 dB (PP4) with a 5 dB increase each time. Average PPI is also shown. The acoustic startle response (ASR) was measured at 110 dB. Welch’s unpaired test (PP1) and Mann–Whitney test (ASR, PP2-PP4 and Average PPI) was applied. Results are expressed as the mean ± SEM. Each circle represents one mouse subject. Male and female data were combined. * *p* value ≤ 0.05, ns = non-significant.

Glucose tolerance (IPGTT) as well as clinical blood biochemistry parameters were similar between variant and WT mice (https://www.mousephenotype.org/impress/index, accessed on 25 July 2023). The auditory brainstem response (ABR) confirmed proper auditory function at 14 weeks of age (https://www.mousephenotype.org/impress/index, accessed on 25 July 2023). Grip strength tests revealed no neuromuscular dysfunction (Figure 2A,B). The pre-pulse inhibition (PPI) test measuring sensorimotor gating also showed similar results for WT and *Atp6v1b2*^emR506*^ HOM mice indicating that HOM mice filter and respond to stimuli in the same way as WT mice (Figure 2C).

**Figure 3:**
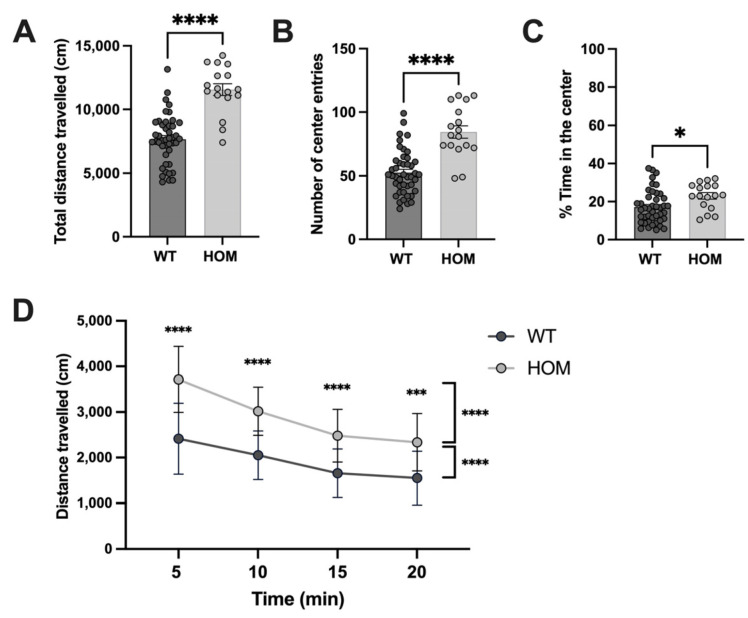
*Atp6v1b2*^emR506*^ HOM mice show locomotion hyperactivity. Total distance travelled (**A**), Number of center entries (**B**), and Time in the center (**C**) during the Open Field assay. Results are expressed as the mean ± SEM. Each circle represents one mouse subject. Male and female data were combined. Student’s unpaired t test was applied. (**D**) Distance travelled over time during the Open Field assay with a 5 min increment. Results are expressed as the mean ± SD. Male and female data were combined. *n* = 44 for WT and *n* = 17 for HOM mice. Two-way ANOVA with RM followed by post-hoc Tuckey’s test for multiple comparisons of the distance travelled over time and post-hoc Sidak’s test for multiple comparisons of distance travelled at each time point were applied. * *p* value ≤ 0.05, *** *p* value ≤ 0.001, **** *p* value ≤ 0.0001.

Locomotor activity of WT and *Atp6v1b2*^emR506*^ HOM was evaluated using the Open Field test. The *Atp6v1b2*^emR506*^ HOM mice travelled longer distances (mean  ±  SEM: WT 7674 ± 292 cm, HOM 11554 ± 460 cm, *p* < 0.0001), entered the center field of the arena more frequently (mean  ±  SEM: WT 52.5 ± 2.7 time, HOM 84.6 ± 4.9 time, *p* < 0.0001)(Figure 3B), and spent more time in the center compared to WT mice (mean  ±  SEM: WT 17.1 ± 1.3%, HOM 23.0 ± 1.7%, *p* = 0.0161)(Figure 3C). Distance travelled over time was also evaluated to see if the mice demonstrated some level of habituation to their environment. Indeed, the distance travelled of *Atp6v1b2*^emR506*^ HOM at any time was significantly longer than WT but like WT mice the distance travelled also decreased significantly over time (Figure 3D). Altogether, the Open Field test results indicates that *Atp6v1b2*^emR506*^ HOM mice present locomotor hyperactivity and tend to have less anxiety-associated behaviors since they spend more time in the center of the arena.

### 3.2. Atp6v1b2^emR506*^ Mice Present Diminished Anxiety-Associated Behaviors

**Figure 4:**
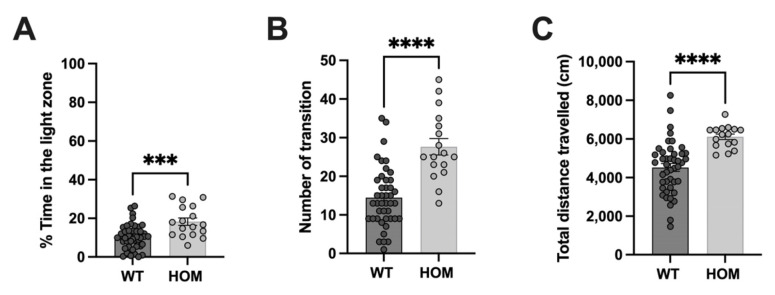
*Atp6v1b2*^emR506*^ HOM mice present less anxiety-like behaviors. Time spent in the light zone (**A**), Number of transitions between light and dark zone, (**B**) and the Total distance travelled (**C**) during the Light-Dark test. Results are expressed as the mean ± SEM. Each circle represents one mice subject. Male and female data were combined. Student’s unpaired t test was applied. *** *p* value ≤ 0.001, **** *p* value ≤ 0.0001.

To further address if *Atp6v1b2*^emR506*^ HOM mice were less anxious, we performed the Light-Dark test. As in the Open Field, *Atp6v1b2*^emR506*^ HOM mice travelled longer distances than the WT mice (mean  ±  SEM: WT 4522 ± 205 cm, HOM 6112 ± 144 cm, *p* < 0.0001) (Figure 4C), once again suggesting that *Atp6v1b2*^emR506*^ HOM mice have locomotor hyperactivity. In addition, *Atp6v1b2*^emR506*^ HOM overall spent more time in the light zone (mean  ±  SEM: WT 10.5 ± 1.0%, HOM 18.3 ± 1.9%, *p* < 0.0001) (Figure 4A) and transitioned more between the two zones (mean  ±  SEM: WT 14.5 ± 1.2 times, HOM 27.7 ± 2.1 times, *p* < 0.0001) (Figure 4B), suggesting again that these mice present less anxiety-related behaviors.

### 3.3. Atp6v1b2^emR506*^ Mice Do Not Have Impaired Contextual or Cued Learning and Memory

**Figure 5:**
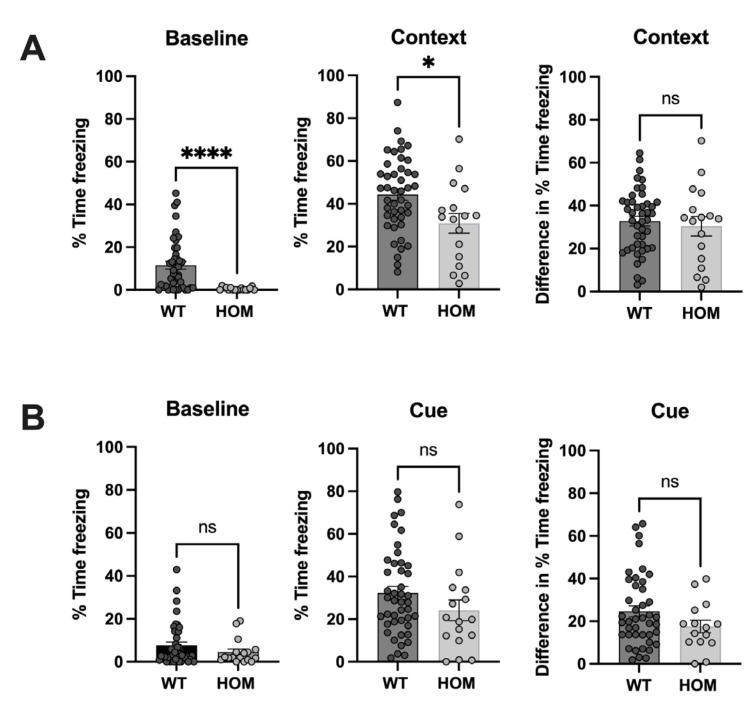
Atp6v1b2 emR506* HOM mice have a normal associative learning/memory. Percentage Time freezing during the contextual (**A**) and the Cue (**B**) segment of the Fear conditioning test. Context and Cue measures were normalized by subtracting the baseline and are represented as the difference in % Time freezing. Results are expressed as the mean ± SEM. Each circle represents one mice subject. Male and female data were combined. Mann–Whitney test (Baselines and Cue—Baseline) and Student’s unpaired t test (Context and Cue) were applied. * *p* value ≤ 0.05, **** *p* value ≤ 0.0001. ns = non-significant.

To address associative learning and memory, mice underwent the fear-conditioning test. At baseline, *Atp6v1b2*^emR506*^ HOM mice froze 23 times less than WT mice (mean  ±  SEM: WT 11.5 ± 1.9%, HOM 0.5 ± 0.2%, *p* = 0.0001) (Figure 5A), consistent with the mice being more active and probably less anxious than WT controls. In the contextual part of the fear-conditioning test, there was no significant difference between WT and *Atp6v1b2*^emR506*^ HOM mice for overall percent time freezing (mean  ±  SEM: WT 32.8 ± 2.2 %, HOM 30.4 ± 4.5%, *p* = 0.5956). As for the cued part of the fear-conditioning test, there were no significant differences between WT and HOM mice at baseline (mean  ±  SEM: WT 7.7 ± 1.5%, HOM 4.6 ± 1.4%, *p* = 0.2863) and comparison of overall percent time freezing showed no significant difference (mean  ±  SEM: WT 24.6 ± 2.6%, HOM 17.6 ± 3.0%, *p* = 0.1801). Altogether, these results suggest that *Atp6v1b2*^emR506*^ HOM have appropriate contextual and cued memory acquisition indicating that they have normal associative learning capacity.

### 3.4. Atp6v1b2^emR506*^ Mice Display Increased Seizure Susceptibility and Stimulus-Induced Seizures

**Figure 6:**
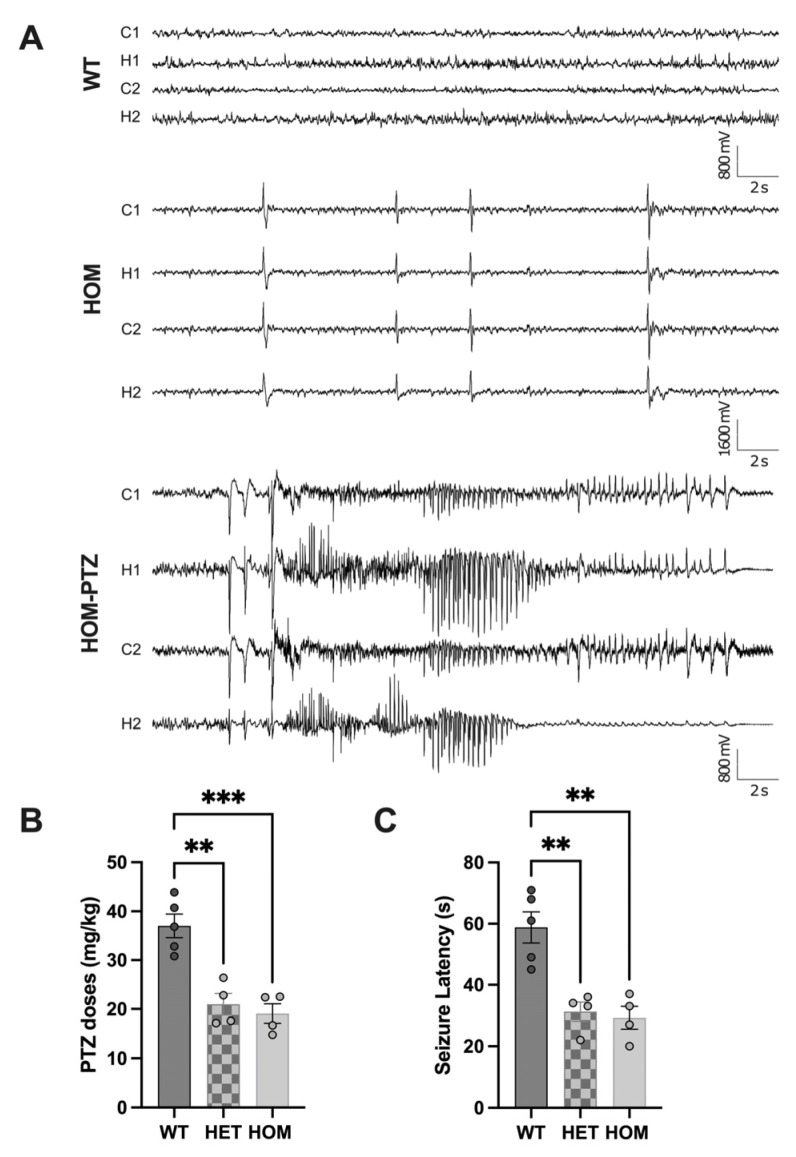
Atp6v1b2 emR506* HOM mice display interictal epileptic activity and a reduced threshold to PTZ-induced seizures. (**A**) Representative examples of EEG traces recorded during wakefulness at baseline in wildtype controls (WT, top) and Atp6v1b2 emR506* HOM mice (HOM, middle), and during a PTZ-induced seizure in a HOM mouse (bottom). (**B**) Quantifications of the minimal dose of PTZ required to induce seizures. (**C**) Quantifications of the latency to the first seizure Results are expressed as the mean ± SEM. Each circle represents one mouse subject. One-way ANOVA followed by post-hoc Sidak’s test for multiple. comparisons were applied. ** *p* value ≤ 0.01, *** *p* value ≤ 0.001.

To assess brain activity and to investigate the presence of epileptic activity in *Atp6v1b2*^emR506*^ mice, we performed continuous intracranial video-EEG recordings in P90-P110 mutant mice and their control WT littermates using bilateral intracranial monitoring in the somatosensory cortex and hippocampus. On 72 h baseline video-EEG recordings, *Atp6v1b2*^emR506*^ HOM mice presented frequent interictal epileptic activity in the form of generalized bilateral synchronous high amplitude isolated spikes or spike-wave complexes (Figure 6A, middle trace), sometimes accompanied by a clinical myoclonic jerk of the trunk and limbs. These events were observed during wake and non-REM sleep in HOM mice and were not in WT mice (Figure 6A, top trace). No spontaneous seizures were recorded in HOM, HET, or WT mice during the 72 h baseline recordings. However, during routine mouse husbandry, *Atp6v1b2*^R506*^ HOM mice displayed stimuli-induced seizures during handling for cage changes (Supplementary Video S1). This also happened during mice phenotyping where 8 *Atp6v1b2*^emR506*^ HOM mice out of 17 had seizures between weeks 11 and 16. No seizures were observed in *Atp6v1b2*^emR506*^ HET mice or WT under similar conditions. 24h video monitoring might have led to the identification of seizures in HET mice or earlier seizures in HOM mice.

To further characterize the seizure susceptibility in our model, we investigated the seizure threshold to PTZ administration (0.01 mg/mL in saline, 0.1 mL/min, i.v. caudal vein). We observed a net reduction in seizure threshold, with lower PTZ doses required to induce seizures in HET (21.0 ± 2.2 mg/kg; *n* = 4) and HOM (19.1 ± 2.0 mg/kg; *n* = 4) p.R506* mice compared to WT controls (37.0 ± 2.4 mg/kg; *n* = 5) (Figure 6B) (One-way ANOVA, *p* = 0.0003). Furthermore, we observed a reduction in seizure latency in HET (31 ± 3 s) and HOM (29 ± 3 s) mutant mice as compared to WT (59 ± 5 s) (Figure 6C) (One-way ANOVA, *p* = 0.0008). These results indicate that the *ATP6V1B2* R506* variant affects network activity and enhances seizure susceptibility to a range of pro-convulsive triggers.

## 4. Discussion

Here we report that *Atp6v1b2*^R506*^ HOM mice have no obvious morphological anomalies apart from being slightly smaller than their WT counterparts. No hearing loss was detected at 14 weeks, in agreement with this phenotype appearing only after 24 weeks of age in previous ATP6V1B2-deficient mouse models [23,28]. Behavioral phenotyping showed that *Atp6v1b2*^emR506*^ HOM mice have locomotor hyperactivity and less anxiety-associated behaviors. The fear conditioning responses suggest that contextual and cued learning and memory is not impaired in *Atp6v1b2*^emR506*^ HOM mice as there were no significant differences in either the contextual or cued part of the test. The percentage of freezing in response to the cued fear conditioning for controls was around 30%. Different protocols yield higher baseline freezing, and the sensitivity of the assay to notice a difference could have been higher if the baseline was increased. The observation that *Atp6v1b2*^emR506*^ HOM mice were freezing less than the WT at baseline is consistent with the mice being more active. Increased locomotor activity in mice can reflect ADHD-associated hyperactivity [29,30,31]. In children with epilepsy, ADHD prevalence varies between 10 to 40% [32,33,34]. Hyperactivity/ADHD have not been reported in individuals with heterozygous p.R506* variant [14]. However, it is possible that the hyperactive phenotype observed in the p.R506* mice reflect more significant network dysfunction in the homozygous condition. It would be interesting to see whether *Atp6v1b2*^emR506*^ HOM mice hyperactivity could be normalized by amphetamine and the pharmacological drug methylphenidate (Ritalin, MPH) used to treat individuals with ADHD. Reduction in hyperactivity using normally psychostimulant drugs is a good indication of an ADHD-associated behavior [30,35,36]. For behavioral assays, we considered males and females together. With higher numbers, we could have analyzed sexes separately to have more granular information, although in humans, there is no noted difference in phenotypes between males and females.

Seizures are an important clinical feature observed in individuals with the p.R506* variant. Although *Atp6v1b2*^emR506*^ HOM mice did not develop spontaneous seizures, handling-induced seizures were observed in 50% of *Atp6v1b2*^emR506*^ HOM mice after the age of 11 weeks. EEG analysis also revealed that *Atp6v1b2*^emR506*^ HOM as well as HET mice, the latter of which may better represent affected individuals in the human population, have a lower seizure threshold to pharmacological triggers compared to WT animals. Pathological variants in ion channels are recurrently and strongly associated with epilepsy [5,37]. Some channelopathies also result in stimulus-induced seizures. *KCNB1* encodes a subunit of the voltage-gated potassium channel K_v_2.1. *KCNB1* p.G379R HOM mutant mice, carrying a variant identified in an infant with developmental and epileptic encephalopathy (DEE), also display hyperactivity and diminished anxiety-associated behaviors together with handling-induced seizures [38]. Notably, only homozygous *KCNB1* p.G379R mice presented such seizures, like in *Atp6v1b2*^emR506*^ HOM mice, while such seizures were not observed in the heterozygous state [38]. This suggests that variants associated with epilepsy in the heterozygous condition in humans are insufficient, in isolation, to induce epilepsy in the heterozygous state in mice, likely reflecting the contribution of other polygenic variants or environmental factors to the net phenotype in human heterozygous.

During this study, the group of Yongui Yuan also published the characterization of genetically modified mice harboring the *ATP6V1B2* p.R506* variant [23,28]. Body weight was also affected, while no other morphological defects were observed. *Atp6v1b2*^R506*^ mutant mice did not show anxiety-associated behaviors in the Open Field, Light-Dark, or in the Elevated Plus Maze (no increase noted). The level of spontaneous activity, or the presence of hyperactivity, was not reported. Learning and memory were assessed using the passive avoidance test (PAT), which is similar to the fear conditioning test. In contrast to our observations, Yuan et al. showed a significant difference between *Atp6v1b2*^R506*^ HOM and WT animals, suggesting that this variant is associated with fear-based behavioral impairment. More experiments will be necessary to address this discrepancy, which may reflect differences in testing paradigm. However, in both studies, the p.506* variant was associated with an epileptic phenotype. Indeed, stimulus-induced seizures were noticed when *Atp6v1b2*^R506*^ HOM mice were placed in the Morris Water Maze [28]. The degree of seizure susceptibility to well-characterized pharmacological triggers or the electrographic phenotype was not reported. Describing seizure threshold to known pharmacological triggers in our model will enable us to further test therapies using a predictable experimental paradigm instead of relying on unpredictable stimulus-induced seizures.

Here we show that the *ATP6V1B2* p.R506* variant in mice is associated with seizures, locomotor hyperactivity, and diminished anxiety-associated behaviors. Moreover, our results confirm that the presence of the p.R506* variant in only one allele is sufficient to increase the susceptibility to seizures. Deeper characterization of the onset of seizures in affected individuals could reveal subtle conditions that trigger epileptic episodes. It would be interesting to know if individuals with the p.R506* variant, or any other deleterious variant in the *ATP6V1B2* gene, are more prone to other types of induced epilepsy or seizures caused by trauma, encephalitis, infections, or fever for example. To conclude, we believe that our mouse model is a valuable tool to study the role of V-ATPases in epilepsy and to screen for new potential drug treatments.

## Data Availability

Data supporting reported results can be found at https://www.mousephenotype.org/impress/index (accessed on 25 July 2023).

## Supplementary Materials

The following supporting information can be downloaded at: https://www.mdpi.com/article/10.3390/genes14081538/s1, Figure S1: Scheme representation of the mice phenotyping pipeline; Video S1: Filming of *Atp6v1b2*^emR506*^ heterozygous mice seizures during cage changes.
