# Supplementary figures and images for: The ATP6V1B2 DDOD/DOORS-Associated p.Arg506* Variant Causes Hyperactivity and Seizures in Mice
Source: Genes (Basel). 2023 Jul 27;14(8):1538. doi: 10.3390/genes14081538 (PMC10454733; doi:10.3390/genes14081538)

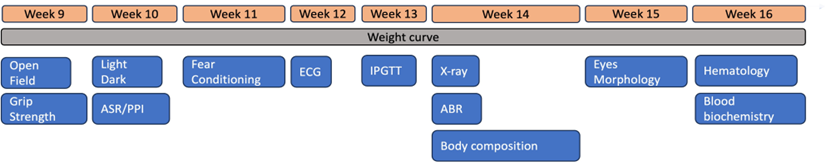

Supplement: Supplementary file 1 [file genes-14-01538-s001.zip › genes-2499064-Figure S1.png]
